# A device specifically designed for the treatment of cerebral venous sinus thrombosis

**DOI:** 10.1093/nsr/nwaf046

**Published:** 2025-02-17

**Authors:** Marc Fisher

**Affiliations:** Beth Israel Deaconess Medical Center, Harvard Medical School, USA

Cerebral venous sinus thrombosis (CVST) is a relatively uncommon cerebrovascular disorder that disproportionally affects younger patients, especially women in the peripartum time period. It can have a devastating outcome [1]. The treatment of CVST is typically done with anticoagulation with a paucity of clinical trial data, but unfavorable outcomes occur in some patients despite this treatment [2]. In patients with acute ischemic stroke secondary to arterial clots, mechanical thrombectomy with a variety of devices has been shown to have a highly beneficial effect in improving outcome with some patients benefiting up to 24 hours after stroke onset [3,4]. Benefit has also been observed in some patients with large ischemic cores treated with this procedure [5]. Selected CVST patients who do not respond to traditional treatment with anticoagulation are potentially candidates for clot removal as is done in arterial stroke patients with a proximal large vessel occlusion. When such procedures are currently performed a device designed to treat arterial clots is employed because devices specifically designed to remove clots from the venous system have not been developed [6]. Using a device designed to remove arterial clots in the venous system can be problematic because of differences between the two vascular systems.

In this manuscript by Li *et al*. [7] the authors describe the development and utilization of a device specifically designed to remove clots from the venous system in the brain. Elegant engineering was utilized to develop a device that can be safely used in the venous system. Computational modeling and an *in-vitro* study were performed to evaluate device performance. It was initially employed in animal studies that demonstrated its effectiveness and safety. Subsequently it was used in 10 CVST patients with good effectiveness in removing venous clots and on the clinical outcomes of these patients.

The development of a device specifically designed for the treatment of CVST is an important step forward in the treatment of this disorder which is uncommon but potentially devastating for afflicted patients. The device will need to be evaluated in a larger sample of CVST patients to determine its safety, ease of use and effectiveness in removing venous clots and improving clinical outcomes. Such studies should be done both in Chinese and non-Chinese populations because safety and clinical responses may vary in different populations. If these studies confirm that this device specifically designed to treat CVST patients is safe and effective the treatment of this disorder will have taken a large step forward. Further studies with this novel device are eagerly anticipated.
